# The Prognostic Significance of Neutrophil-to-Lymphocyte Ratio and Platelet-to-Lymphocyte Ratio for Long-Term Survival in Patients With Severe Left Ventricular Dysfunction and Implantable Cardioverter Defibrillator

**DOI:** 10.7759/cureus.47441

**Published:** 2023-10-21

**Authors:** Mustafa Ozan Çakır

**Affiliations:** 1 Cardiology, Zonguldak Bülent Ecevit Üniversitesi Faculty of Medicine, Zonguldak, TUR

**Keywords:** systolic heart failure, ventricular ejection fraction, implantable cardioverter-defibrillators, lymphocytes, mortality

## Abstract

İntroduction: Systemic inflammation resulting from comorbidities such as arterial hypertension, diabetes, and obesity is responsible for the pathogenesis of myocardial structural and functional changes in heart failure. The neutrophil-to-lymphocyte ratio (NLR) and platelet‐to‐lymphocyte ratio (PLR) are novel biomarkers of inflammation. The aim of this study was to evaluate the relationship between blood NLR and PLR levels and one-year cardiac mortality in primary prevention patients with left ventricular ejection fraction (LVEF) ≤35%, using an intracardiac defibrillator.

Methods: A total of 180 compensated heart failure patients with LVEF<35% (ischemic or nonischemic) and implantable cardioverter-defibrillator (ICD) therapy for primary prevention who applied to the cardiology outpatient clinic of Zonguldak Bülent Ecevit Üniversitesi Hospital, Zonguldak, Türkiye, between March 2018 and June 2019 were consecutively included. The patients were followed for one year after the application.

Results: In the multivariate logistic regression analysis model, only NLR (OR: 1.328; 95%CI: 1.129-1.563; p <0.01) was found independently associated with the risk of one-year cardiovascular mortality. Based on the NLR, levels were 2.69 ng/ml, and the area under the curve was found to be 0.795 (95%CI: 0.729-0.862) in the evaluation made with the receiver operating characteristic curve.

Conclusion: High NLR ratio levels independently predicted one-year cardiac mortality in patients with LVEF<35% and ICD for secondary protection. Large-scale randomized studies are needed to fully demonstrate the relationship between NLR levels and cardiovascular mortality in patients with severe left ventricular dysfunction and ICD.

## Introduction

Heart failure (HF) prevalence in the United States is expected to rise to more than eight million by 2030 [1]. HF remains the primary cause of hospitalization in the elderly, and mortality rates remain high despite significant advances in targeted therapy approaches [2]. The five-year survival rate for patients with HF is less than 50% [3]. HF is a complex and sophisticated syndrome that involves various pathological mechanisms that contribute to the formation of structural or functional abnormalities, ultimately leading to an imbalance between cardiac capacity and peripheral oxygen demand [4]. Systemic inflammation resulting from comorbidities such as arterial hypertension, diabetes, and obesity is responsible for the pathogenesis of myocardial structural and functional changes in HF [5]. Various inflammatory biomarkers are linked with disease severity and prognosis of HF [6].

Complete blood count (CBC) is a cost-effective and readily available test that is frequently used in daily practice. White blood cell (WBC) count, which is a typical marker of inflammation, is examined within the CBC test. Neutrophilia is related to increased hospital admission and mortality due to HF [7], and lymphopenia is an indicator of poor prognosis in patients with HF [8,9]. The neutrophil-to-lymphocyte ratio (NLR) and platelet‐to‐lymphocyte ratio (PLR) are novel biomarkers of inflammation [10,11]. They are elevated in various cardiovascular diseases and can also be used as prognostic signs of HF patients [12-17].

The data on the association between blood NLR or PLR levels and the prognosis in patients with heart failure with left ventricular ejection fraction (LVEF) <35% are limited. In the current study, we aimed to evaluate the relationship between blood NLR and PLR levels and one-year cardiac mortality in primary prevention patients with LVEF ≤35%, who are using an implantable cardioverter defibrillator (ICD).

## Materials and methods

Study population

In this prospective study, a total of 180 compensated HF patients with LVEF< 35% (ischemic or nonischemic heart disease) and ICD therapy for primary prevention who applied to the cardiology outpatient clinic of Zonguldak Bülent Ecevit Üniversitesi Hospital, Zonguldak, Türkiye, between March 2018 and June 2019 were consecutively included. The study was conducted in accordance with the principles of Good Clinical Practice and the Declaration of Helsinki. Clinical Research Ethics Committee of the Zonguldak Bülent Ecevit Üniversitesi Faculty of Medicine gave the ethical approval (approval number: 2018-45-31/01). Informed consent was obtained from all the patients.

The exclusion criteria involved age <18 years, acute ST-elevation myocardial infarction (STEMI) and non-STEMI (NSTEMI) at admission, severe valve disease (aortic stenosis, aortic regurgitation, mitral stenosis, or mitral regurgitation), end-stage kidney disease requiring hemodialysis, advanced liver disease, acute and/or chronic lung disease, cardiac arrhythmia other than atrial fibrillation, life expectancy <6 months due to noncardiac diseases, and previous heart transplantation.

In addition, patients affected by active or chronic inflammatory or autoimmune diseases, presenting active or past hematological proliferative diseases or oncological history, patients who received blood transfusions, those who were treated with anti-inflammatory drugs (systemic steroids, immunosuppressive drugs) in the last six months, or patients who were having active infection were excluded.

Information about patient demographics, symptoms and signs of HF, medical history, ECG and echocardiography, laboratory test results, and current medication were collected at index admission. Medical treatment of the patients was optimized according to the 2016 European Society of Cardiology (ESC) HF guideline at admission.

Laboratory data

Laboratory data blood sampling was done from the antecubital vein at admission. Blood samples were immediately sent to the laboratory for analysis. Ethylenediamine-tetraacetic acid (ETA)-containing tubes were used for the hemogram assessment. A CBC test, including differentials, was evaluated using an automated blood cell counter (Coulter LH 780 Hematology Analyzer; Beckman Coulter, Inc., Brea, California, United States). Blood samples were also used to measure serum levels of electrolytes, creatinine, blood urea nitrogen, N-terminal pro-B-type natriuretic peptide (NT-proBNP), and C‐reactive protein (CRP) levels.

Echocardiographic analysis

Echocardiography was performed on all patients. The VIVID 7 Cardiovascular Ultrasound System (GE Vingmed Ultrasound AS, Horten, Norway) with a 3.5 MHz probe was used for echocardiographic evaluation with a 3.5 MHz probe. Echocardiographic measurements were made in the left supine position. Parasternal long and short axis, apical angle standard viewing windows were used. The ejection fraction was calculated by the modified Simpson's method. All echocardiographic images were evaluated by an experienced cardiologist.

Study endpoints and definitions

NLR was calculated by dividing absolute neutrophil count to lymphocyte count and PLR was calculated by dividing absolute neutrophil counts to platelet counts using the same blood samples. Estimated glomerular filtration rate (eGFR) was calculated with the help of the equation given in the modification of the diet in renal disease (MDRD) study [18]. Patients with incomplete medical records were disqualified. The patients were followed for one year after the application to the outpatient clinic with telephone calls at monthly intervals. Follow-up face-to-face visits were scheduled at three, six, nine, and 12 months. The long-term all-cause mortality was the primary outcome in patients with LVEF<35%.

Statistical analysis

The conformity of the variables to the normal distribution was examined using visual (histogram and probability graphs) and analytical (Kolmogorov-Smirnov/Shapiro-Wilk) tests. Descriptive analyses were given using the median (minimum-maximum) for non-normally distributed variables. Categorical data were given using frequency (n) and percentage (%). The Mann-Whitney U test was used for non-normally distributed variables. A comparison of categorical variables between groups was made using the chi-square test and Fisher's exact test as appropriate. Clinical parameters that were analyzed at p <0.20 level were entered into univariate logistic regression analyses. Univariate logistic regression analyses were performed to estimate the proportion of possible risk factors for the all-cause mortality of patients with LVEF<35%. Clinical parameters that were analyzed at p<0.20 in a univariate model were entered into multivariate logistic regression analyses for defining independent risk factors related to mortality.

In the current study, p< 0.20 was found in a univariate model for LVEF, %, BMI, high-sensitivity troponin T (hsTnT), NLR, and PLR. Multivariate logistic regression analyses were performed for LVEF, %, BMI, hsTnT, NLR, and PLR. The backward stepwise method was used to select the most suitable model. OR with 95%CI were established for each variable and p <0.05 was considered significant for all comparisons. Receiver operating characteristic (ROC) curves matching the selected logistic regression models were constructed, and the areas under the curve (AUC) or C-statistic were calculated. Statistical analyses were performed using the IBM SPSS Statistics for Windows, Version 20.0 (Released 2011; IBM Corp., Armonk, New York, United States).

## Results

A total of 180 patients met the criteria and were included; 57% (n=102) of the patients were female and the median age was 75 (Interquartile range (IQR): 67-80). Comorbidities present in the patient population included hypertension (69%; n=125), diabetes mellitus (44%; n=79), coronary artery disease (21%; n=37), atrial fibrillation (7%; n=12), chronic obstructive pulmonary disease (74%; n=41), and dyslipidemia (35%; n=63). The median LVEF of the patients was found to be 32 (IQR 24-32) (Table 1).

**Table 1:** Baseline demographics Values are given as median (1st quartile-3rd quartile) or n (%) eGFR: estimated glomerular filtration rate; BMI: body mass index; hsTnT:  high‐sensitivity troponin T; hs‐CRP; high‐sensitivity C‐reactive protein; NT‐proBNP: N-terminal pro-B-type natriuretic peptide; IQR: interquartile range

Baseline demographics (n= 180)	Values
Age (years), Median (IQR)	75 (67-80)
Female, n (%)	102 (57)
Left ventricular ejection fraction (%), Median (IQR)	32 (24-32)
Coronary artery disease, n (%)	37 (21)
Hypertension, n (%)	125 (69)
Diabetes mellitus, n (%)	79 (44)
Cerebrovascular accident, n (%)	13 (7)
Dislipidemia, n (%)	63 (35)
Atrial fibrillation, n (%)	12 (7)
Chronic obstructive pulmonary disease, n (%)	74 (41)
eGFR, Median (IQR)	80 (75-81)
BMI (kg/m²), n (%)	35 (16)
NT‐proBNP (pg/mL), Median (IQR)	740 (593-1065)
hsTnT (ng/L), Median (IQR)	25 (11-36)
hs‐CRP (mg/L), Median (IQR)	1.64 (1.20-2.20)
Neutrophil‐to‐lymphocyte ratio, Median (IQR)	2.4 (1.6-4.0)
Platelet‐to‐lymphocyte ratio, n (%)	35 (17)

After a follow‐up period of 365 days, 37 patients died; 33 of these deaths were attributed to cardiac causes (exacerbation of HF, n=15; fatal arrhythmia or sudden cardiac death, n=11; myocardial infarction, n=4; and other causes of death, n=3) and four were linked due to non-cardiac causes (infection, n=1; renal failure, n=1; and other causes of death, n=2).

When the patients were classified in terms of one-year cardiac mortality, no statistically significant difference was found in terms of age (p=0.90), gender (p=0.79), hypertension (p=0.79), atrial fibrillation (0.88), diabetes mellitus (p=0.56), coronary artery disease (p=0.40), ejection fraction (p=0,11), or chronic obstructive pulmonary disease (p=0.38) (Table 2). Patients who died in one year had higher serum hsTnT levels(p<0.01), NLR (p<0.01), and PLR (p<0.01).

**Table 2:** Baseline characteristics of patients according to mortality rates Numerical variables were presented as the mean ± standard derivation or median (1st quartile-3rd quartile), and categorical variables were defined as n (%) eGFR: estimated glomerular filtration rate; BMI: body mass index; hsTnT: high‐sensitivity troponin T; hs‐CRP: high‐sensitivity C‐reactive protein; NT‐proBNP: N-terminal pro-B-type natriuretic peptide; IQR: interquartile range

Characteristics	Survived, (n=147)	Died, (n=33)	p-value
Age (years), mean± SD	73.1 ± 9.5	73.4 ± 8.7	0.90
Female gender, n (%)	84 (57.1%)	18 (54.5%)	0.79
Hypertension, n (%)	125 (85%)	19 (87.9%)	0.79
Left ventricular ejection fraction (%), mean± SD	28.8 ± 6.1	27.0 ± 6.3	0.11
Diabetes mellitus, n (%)	63 (42.9%)	16 (48.5%)	0,56
Cerebrovascular accident, n (%)	10 (6.8%)	3 (9.0%)	0.24
Coronary artery disease, n (%)	32 (21.8%)	5 (15.2%)	0.40
Chronic obstructive pulmonary disease, n (%)	32 (38.8%)	32 (51.5%)	0.38
eGFR, mean± SD	78.1 ± 8.4	77.2 ± 8.6	0.58
Atrial fibrillation, n (%)	10 (6.8%)	2 (6.1%)	0.88
Dislipidemia, n (%)	26 (66.0%)	139 (60.6%)	0.56
BMI (kg/m²), mean± SD	26.4 ± 3.7	25.4 ± 3.0	0.15
NT‐proBNP (pg/mL), median (IQR)	740 (594-1265)	740 (530-1035)	0.31
hsTnT (ng/L), median (IQR)	15 (6-28)	20 (14-41)	0.03
hs‐CRP (mg/L), median (IQR)	1,4 (1.3-2.1)	1,8 (1.3-2.5)	0.09
Neutrophil‐to‐lymphocyte ratio, median (IQR)	2.0 (1.5-3.4)	3.7 (2.8-6.2)	<0.01
Platelet‐to‐lymphocyte ratio, median (IQR)	112.8 (81.7-147.4)	140.8 (111.0 -188.6)	<0.01

Univariate logistic regression analyses were performed to estimate the proportion of possible risk factors for cardiac death. Clinical parameters that were analyzed at p<0.20 level in a univariate model were entered into multivariate logistic regression analyses for defining independent risk factors related to cardiac mortality. In the univariate regression analysis of possible risk factors, LVEF, BMI, hsTnT, NLR, and PLR were found to be associated with the risk of mortality (p<0.20).

In the multivariate logistic regression analysis model including BMI, hsTnT, NLR, and PLR, only NLR (OR: 1.328; 95%CI: 1.129-1.563; p<0.01) was found independently associated with the risk of one-year cardiovascular mortality (Table 3). In the multivariate model, PLR (OR: 0.995; 95%CI: 0.985-1.005; p=0.29) was not related statistically to long-term mortality. Serum hsTnT and PLR levels tended to be high in the cardiac death group (15.0 ng/L vs 19.5 ng/L and 112.8 vs 140.8, respectively). However, this finding was not statistically significant in multivariate analyses for serum hsTnT (p=0.07) or PLR (p=0.29) (Table 3).

**Table 3:** Univariate and multivariate logistic regression analyses demonstrating independent predictors of mortality LVEF: left ventricular ejection fraction; CI: confidence interval; OR: odds ratio; BMI: body mass index; hsTnT: high‐sensitivity troponin T

Parameters	Univariate Analyses		Multivariate Analyses	
	OR (95%CI)	p-value	OR (95%CI)	p-value
Left ventricular ejection fraction (%)	0.957 (0.903–1.015)	0.14	0.945 (0.874–1.021)	0.15
BMI (kg/m²)	0.923 (0.828–1.028)	0.15	0.914 (0.840–1.044)	0.19
hsTnT (ng/L)	0.973 (1.003–1.007)	0.06	1.003 (1.000–1.007)	0.07
Neutrophil‐to‐lymphocyte ratio	1.328 (1.129–1.563)	<0.01	1.555 (1.143-2.116)	<0.01
Platelet‐to‐lymphocyte ratio	1.008 (1.003–1.014)	<0.01	0.995 (0.985–1.005)	0.29
hs‐CRP (mg/L)	0.994 (0.912–1,082)	0.89	-	-

**Figure 1:**
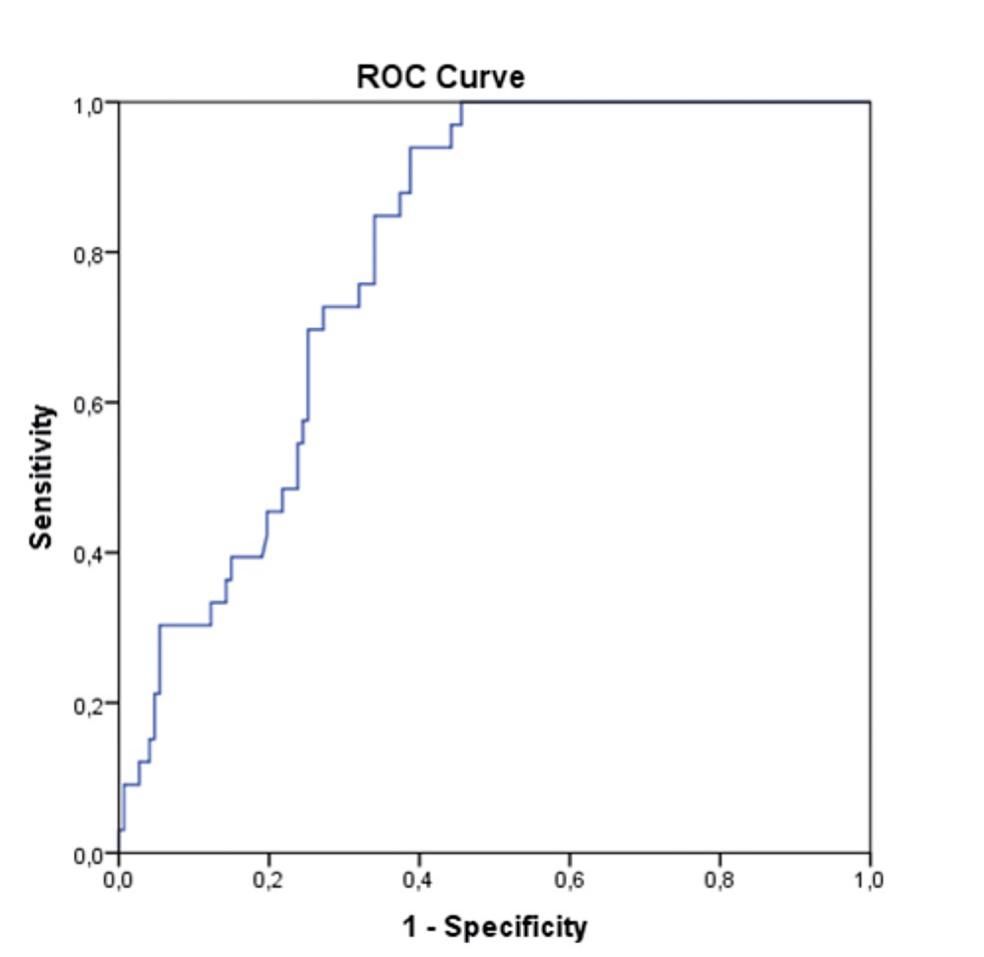
Receiver operating characteristic (ROC) curve representing the cut-off point of neutrophil‐to‐lymphocyte ratio in prediction of cardiac death

ROC curve results are illustrated in Figure 1. The c-index based on area under the ROC curve (AUC) values for the NLR levels in predicting one-year cardiovascular mortality was calculated as 0.795 (95%CI: 0.729-0.862). The optimal cut-off point predicting long-term cardiac death in the study group according to NLR levels was 2.69 ng/ml with 0.85 sensitivity and 0.66 specificity.

## Discussion

As a result of this study, the presence of clinical and laboratory parameters that can predict long-term cardiac mortality risk in patients with compensated HF and ICD was investigated. The major finding of our study is that NLR, a simple inflammatory marker, can predict future long-term mortality in patients with low ventricular ejection fraction and their mortality risk. PLR was associated with the development of cardiac mortality in univariate analyses but in multivariate regression analyses, it was not found to be an independent risk factor for increased long-term mortality.

In the innate immune system, neutrophils represent the acute phase of the inflammatory response, whereas lymphocytes are involved in the adaptive immune system and promote the induction of autoimmune inflammation in the chronic inflammatory response. With this mechanism, the NLR combines the innate and adaptive immune systems, making NLR an interesting biomarker of inflammation [18,19].

Higher levels of NLR cause the downregulation of many complement component genes. Individuals with high NLR are suspected to be suffering the loss of major cardiovascular protective mechanisms provided by their complement systems [20].

There is more evidence in the literature demonstrating the value of NLR as a prognostic marker in cardiovascular disease. In a study conducted by Uthamalingam et al. on 1200 individuals with an average follow-up period of 26 months, it was observed that higher NLR levels were significantly related to all-cause mortality in acute AF [21]. Similarly high NLR levels cause an increased risk of major adverse cardiovascular events (MACE), especially in elderly patients with chronic HF [22]. NLR or PLR counting are easily accessible and affordable tests. Another advantage of NLR and PLR as compared to NT‐proBNP or CRP is that they are not affected by some physiological comorbidities such as dehydration and exercise.

A standardized cut-off value for NLR has not been determined in the literature. In limited studies, NLR levels were commonly classified as quartiles rather than standard values, and a generally fixed cut-off value could not be obtained. Therefore the routine usage of NLR for determining the prognosis of HF in clinical practice is limited. Randomized controlled studies are needed in a larger patient group in order to determine prognosis so that NLR can be used as a standard in the clinic.

Also, the plasma NLR and PLR levels are linked with an increased number of comorbidities in patients with HF with preserved ejection fraction [23]. Elevated platelet count by itself is an inflammatory marker related to worse cardiovascular outcomes and PLR expresses the association between prothrombotic and inflammatory status [24,25]. Previous reports in the literature show that PLR levels (either in combination with NLR or separately) are associated with short or long-term mortality in acute or chronic HF [23,26,27]. We found no relationship between PLR and long-term cardiac mortality in this study.

B-type natriuretic peptide (BNP)/NT‐proBNP is known as an important marker for predicting prognosis in patients with HF [28,29]. However, we did not find a relationship in terms of cardiac mortality. Also, plasma CRP levels were not associated with long-term cardiac mortality.

To the best of our knowledge, this is the first prospective study to examine the relationship between plasma NLR and PLR levels and one-year cardiovascular mortality in HF patients with LVEF<35% who had an ICD implant for secondary protection. There are several limitations of our study. First, it was a single-center study with a limited sample size. Secondly, in the study, only NLR and PLR levels were examined at the time of admission; there is a possibility that the blood values of these biomarkers may have changed in some patients during the follow-up period. Third, the causes of cardiac deaths were not analyzed in depth, only all-cause deaths were evaluated.

## Conclusions

High NLR levels independently predict one-year cardiac mortality in patients with LVEF<35% who had an ICD for secondary protection. Large-scale randomized studies are needed to fully demonstrate the relationship between NLR levels and cardiovascular mortality in patients with LVEF <35% and to observe the possible prognostic effects of anti-inflammatory treatments in preventing cardiovascular mortality.
